# The First 24 h Hemodynamic Management in NICU after Revascularization Surgery in Moyamoya Disease

**DOI:** 10.1155/2021/5061173

**Published:** 2021-10-12

**Authors:** Jie Song, Yu Lei, Long Chen, Chao Gao, Wei Ni, Xing Wu, Gang Wu, Ying Mao, Jin Hu, Yuxiang Gu

**Affiliations:** Department of Neurosurgery, Huashan Hospital, Fudan University, 12 Wulumuqi Zhong Road, Shanghai 200040, China

## Abstract

**Objective:**

To evaluate whether hemodynamic factors are risk factors for prognosis in moyamoya disease (MMD).

**Materials and Methods:**

The retrospective study reviewed a single-center MMD cohort in Huashan Hospital from August 2017 to January 2020. Stroke events in 30 days and follow-up modified Rankin Scale (mRS) grade were recorded. Systematic assessments with perioperative mean arterial pressure (MAP), red blood cell (RBC) parameters, and fluid management were also conducted. Logistic regressions were applied to evaluate the predictors of worse outcomes. Data was analyzed using SPSS 24.0.

**Results:**

Admission to neurological intensive care unit (NICU) totalled about 347 after revascularization surgery. The result showed that the higher the postoperative MAP level (favorable group 95.7 ± 11.4 mmHg vs. unfavorable group 103.6 ± 10.4 mmHg, *p* < 0.001) and the greater the MAP variability (favorable group 0.26 ± 13.2 vs. unfavorable group 7.2 ± 13.5, *p* = 0.006) were, the higher the patient's follow-up mRS grade was. What is more, a higher early postoperative Hb level also seemed to predict a worse long-term clinical outcome (favorable group 116.9 ± 17.1 g/L vs. unfavorable group 123.7 ± 13.0 g/L, *p* = 0.03), but the difference disappeared after adjusting sex and age. Logistic regression analyses showed that a higher level of postoperative MAP (*β* = 0.024, 95% CI (0.004, 0.044), and *p* = 0.02) within the first 24 h in NICU might be the short-term risk factor. For long-term outcome, a higher level (*β* = 1.058, 95% CI (1.022, 1.096), and *p* = 0.001) and a greater variability (*β* = 30.982, 95% CI (2.112, 454.414), and *p* = 0.01) of postoperative MAP might be the negative predictors of mRS grade.

**Conclusions:**

The early postoperative hemodynamic management might be extremely critical for patients with MMD. Both high postoperative MAP levels and large MAP variability might affect the prognosis. What is more, we also found that a higher postoperative Hb level might be related with a worse outcome.

## 1. Introduction

Moyamoya disease (MMD) is characterized by the formation of an abnormal vascular network at the base of the brain, which could result in hemodynamic compromise significant different from normocapnia [1, 2]. Owing to the inadequacy of collateral sources of blood flow, patients with MMD often suffer from reduction of perfusion pressure, insufficiency of collateral blood supply, and ultimately decrease of blood flow in the distal territory [1–4]. Revascularization surgery is an effective surgical procedure to improve the steno-occlusive lesions, reconstruct cerebral blood supply system, and prevent the occurrence of long-term complications [4, 5]. Currently, superficial temporal artery-middle cerebral artery (STA-MCA) anastomosis with or without indirect bypass is generally used as the standard surgical treatment for MMD [1, 2, 6–8].

Efforts have been made to modify surgical modalities for better effect and fewer complications, but revascularization surgery of MMD may instantly aggravate the chaotic state of cerebral blood flow, causing acute stroke or hyperperfusion syndrome [4, 9–12]. During the perioperative period, the type of anesthetic drug, fluid resuscitation, blood loss and hematocrit (Hct), end tidal carbon dioxide (EtCO2), temperature regulation, urine output, and the type of surgical procedure being done may affect cerebral blood flow and cerebral perfusion pressure [8, 12–14]. Under these conditions, inadequate cerebral perfusion may augment the risk for the occurrence of cerebral infarctions in MMD with middle cerebral artery stenosis or carotid artery occlusion [13, 15, 16]. Thus, perioperative conditions other than the surgery itself are of equal importance and are issues needed to be addressed with necessity [11, 17, 18]. A standardized protocol for the perioperative management will benefit patients with MMD and improve their short- and long-term prognosis after surgery.

Throughout the entire perioperative period, early postoperative period is particularly critical, in which intensive care is generally required [1, 5, 18, 19]. Literatures have shown that surgical complications of MMD are largely related to early postoperative management, and the hemodynamic changes in this period have the greatest impact on the prognosis [5, 18, 19]. It is speculated that there is a transient change in cerebral blood flow in the early postoperative period, and the cerebrovascular autoregulation has not fully recovered, and the ability to resist hemodynamic changes is poor [9, 10, 20]. However, it is still lacking in the researches about the hemodynamic management of MMD patients in the first 24 h in neurological intensive care unit (NICU) after revascularization surgery. In this study, we retrospectively analyzed the perioperative hemodynamic parameters of patients with MMD who received operations in our institute over last 3 years and aimed to evaluate whether perioperative hemodynamic changes are risk factors for short-term and long-term prognosis.

## 2. Materials and Methods

### 2.1. Participants

The participants enrolled in this study were a single-center cohort of patients with MMD treated at Huashan Hospital from August 2017 to January 2020. Written informed consents were obtained from all of the participants and/or their guardians at admission. The diagnosis of MMD was based on the criteria issued by the Japanese Ministry of Health, Labor, and Welfare [7]. Moyamoya syndrome was defined as a secondary moyamoya phenomenon caused by severe traumatic brain injury, head and neck irradiation, hyperthyroidism, or autoimmune disease [7]. The inclusion criteria were as follows: (1) age between 18 and 80 years; (2) MMD or moyamoya syndrome confirmed by digital subtraction angiography (DSA); (3) patients who underwent revascularization surgery; (4) after operations, patients were monitored in NICU for at least 24 hours; (5) patients who were followed up for more than six months; and (6) patients with complete information and follow-up data. We excluded patients with intracranial aneurysms or other cerebrovascular diseases. Patients with moyamoya syndrome caused by Down's syndrome, systemic vasculitis, neurofibromatosis, sickle cell disease, history of irradiation, or hyperthyroidism were also excluded [6]. The study was approved by the Huashan Hospital Research Ethics Committee.

Operations were performed by the surgeons who perform more than 200 bypass surgeries each year between August 2017 and January 2020. The surgical indications were in accordance with Japanese guidelines for diagnosis and treatment of MMD [7, 21]. The patency of bypass graft was confirmed by intraoperative indocyanine angiography routinely.

### 2.2. NICU Hemodynamic Management

#### 2.2.1. Blood Pressure (BP) Management

BP control mainly depended on patients' baseline BP level and their clinical manifestations. Before revascularization surgery, the systolic BP (SBP) was maintained under 150 mmHg for all the patients, and diastolic blood pressure (DBP) was maintained under 90 mmHg. During the operation, BP was closely monitored through invasive arterial monitoring. The SBP was strictly controlled within the normal range. After patients recovered from the general anesthesia, they were transferred to the NICU and BP was measured every half an hour through noninvasive monitor. For patients with hypertension, SBP was maintained at 130~140 mmHg with the help of intravenous antihypertensive drugs. Otherwise, SBP was controlled at the baseline level.

Mean arterial pressure (MAP) and MAP variability were defined as the following formulas:

(1) $$\begin{array}{c} \text{MAP}=\text{DBP}+\frac{1}{3}\left(\text{SBP}-\text{DBP}\right),\\ \text{MAP variance}=\frac{{\text{SBP}}_{\text{post}}-{\text{SBP}}_{\text{pre}}}{{\text{SBP}}_{\text{pre}}}. \end{array}$$

#### 2.2.2. Fluid Management

Generally, patients with MMD were in a state of fasting for at least 8 h before general anesthesia. To avoid the possible loss of extracellular fluid, each patient was given an intravenous administration of 5% glucose saline or Ringer lactate solution (1000~1500 ml) before anesthesia. During the operation, each patient would be given 1500~2000 ml intravenous fluid routinely. When transferred to NICU after surgery, sufficient fluid resuscitation was performed and all of the patients would be given another 1500~2000 ml intravenous fluids during the first 24 h. It should be noted that dehydrating agents such as mannitol were routinely used to prevent brain edema caused by surgery. Those agents were not included in the intravenous fluid we mentioned.

### 2.3. Neuroimaging Assessment

All of the patients included in our study underwent head computed tomography (CT), magnetic resonance imaging (MRI), DSA, and diffusion-weighted imaging (DWI) within seven days before operations. For all patients, CT scans were acquired immediately after and 24 h after revascularizations to determine whether there was any intracranial hemorrhage and/or newly developed cerebral infarction. Whenever patients had newly developed symptoms, CT or MRI scans were performed at once. During the early postoperative period, CT angiography was not performed routinely. Instead, the patency of the bypass was calculated by DSA six months after combined revascularization. Postoperative newly developed cerebral infarction was defined as a low-density lesion on CT scans, a high-signal-intensity lesion on DWI images, and a low-signal intensity lesion on the apparent dispersion coefficient images.

### 2.4. Follow-Up

In accordance with previous literatures, short-term phase was defined as <30 days after revascularization [1]. All of the patients with MMD were followed up by telephone, text messaging, and/or clinical services. Clinical outcomes were recorded during follow-up, including recurrent strokes (intracranial hemorrhage or cerebral infarction after revascularization surgery), neurological status, and radiological data. If required, patients received contralateral revascularization during follow-up. For the purpose of statistical analysis, short-term outcomes were classified into 3 grades: (1) grade 1: no symptom, no stroke; (2) grade 2: symptoms onset, no stroke; and (3) grade 3: symptoms onset with stroke. A modified Rankin scale (mRS) was used to determine the neurological function outcome. Long-term outcomes were dichotomized into 2 groups accordingly: favorable group: mRS 0–2 and unfavorable group: mRS 3-5 [22].

### 2.5. Statistical Analysis

All statistical analyses were performed with SPSS 24.0. Demographic and baseline data and perioperative hemodynamic characteristics were compared according to the short-term and long-term prognosis groups. The Fisher exact test, the *χ*^2^ test, and the Student *t*-test were used as appropriate. Data were presented as mean ± standard deviation (SD), median and range, or percentage. Logistic regression models were constructed to evaluate the association between short-term and long-term outcomes (independent variables) and perioperative MAP, red blood cell (RBC) parameters, and fluid transfusion (dependent variables), adjusting age and sex. *p* < 0.05 is the cut-off point.

## 3. Results

Admission to NICU totalled about 347 after revascularization surgery, including 237 patients with unilateral and 55 patients with bilateral revascularization. Among them, 255 patients were diagnosed with MMD and 37 patients were diagnosed with moyamoya syndrome (36 unilateral and 1 bilateral). The baseline characteristics of patients are summarized in Table 1. Among these patients, 87 patients presented with cerebral hemorrhage and 229 patients presented with ischemic symptoms as the initial presentation. 303 underwent STA-MCA anastomosis (87.3%), and EDMS were applied in 44 patients (12.7%) under general anesthesia. The follow-up period ranged from 10 months to 31 months. Patients' average mRS score is 0.7 ± 1.10 (Table 1).

Table 2 compares the preoperative and immediate postoperative variables, respectively. After revascularization surgery, the higher the MAP level (favorable group 95.7 ± 11.4 mmHg vs. unfavorable group 103.6 ± 10.4 mmHg, *p* < 0.001) and the greater the MAP variability (favorable group 0.26 ± 13.2 vs. unfavorable group 7.2 ± 13.5, *p* = 0.006) were in the first 24 h in NICU, the higher the patient's long-term mRS grade was. What is more, a higher early postoperative Hb level also seemed to predict a worse long-term clinical outcome (favorable group 116.9 ± 17.1 g/L vs. unfavorable group 123.7 ± 13.0 g/L, *p* = 0.03), but the difference disappeared after adjusting sex and age. However, a simple statistical comparison did not find that perioperative hemodynamic parameters had any significant effect on the stroke events in 30 days in MMD.

Logistic regression analyses were further executed. For short-term outcome, a higher level of postoperative MAP (*β* = 0.024, 95% CI (0.004, 0.044), and *p* = 0.02) within the first 24 h in NICU might be the risk factor for poor prognosis (Table 3), even after adjusting confounding factors (age and sex). For long-term outcome, the regression analysis indicated that a higher level (*β* = 1.058, 95% CI (1.022, 1.096), and *p* = 0.001) and a greater variability (*β* = 30.982, 95% CI (2.112, 454.414), and *p* = 0.01) of postoperative MAP might be the predictors of unfavorable mRS grade as shown in Table 3.

## 4. Discussion

This is the first study with a large sample size to obtain the intensive hemodynamic management of MMD within the 24 h in NICU after revascularization surgery. We found that both high MAP levels and large MAP variability might affect patients' short-term or long-term prognosis. Thus, it is important to strictly control postoperative MAP within normal range and keep it as the preoperative level. What is more, in the first 24 h in NICU, a higher Hb level might result in a worse mRS grade during follow-up.

Based on our results, higher MAP levels and greater variability were independently associated with unfavorable outcomes in MMD. The underlying mechanism of higher MAP levels might be that fragile collateral vessels cannot tolerate transient changes in cerebral blood flow after revascularization [23]. Thus, a higher level of MAP might lead to intracranial hemorrhage and resulted in devastating consequences. In 2014, Kazomata et al. have also demonstrated that the immediate postoperative intracerebral hemorrhage was associated with a high level of pre- and postoperative BP, especially about systolic BP [12]. Besides, to overcome chronic cerebral hypoperfusion and hypoxia, patients with MMD generally have compensated partially by increasing regional cerebral blood volume and regional oxygen extraction and the cerebrovascular autoregulation have impaired [4, 5, 12, 15, 18, 23]. Bypass surgery theoretically makes patients more vulnerable to an unstable BP, and patients were more likely to suffer from adverse events [5, 15, 18, 23]. Another hazard of higher MAP level is that it may lead to cerebral hyperperfusion syndrome (CHP), which is not rare in postoperative patients with moyamoya disease [11].

A great many reports have confirmed the adverse of BP variability [24–28], but rare studies have focused on the patients with MMD. It seems plausible to understand its relationship with long-term outcomes. Reasons are as follows: (1) BP variability can aggravate the severity because recurrent sudden rises/falls of BP are the contributor of hematoma expanding or ischemia areas increasing [24–26]. (2) BP fluctuations can amplify the secondary brain injury within the potentially viable perihematomal region by directly influencing CBF and CPP [26, 27]. (3) BP rise and fall can disrupt the blood-brain barrier, promote vasogenic brain edema, and cause brain cell death [26, 27]. As for BP was monitored in NICU every half an hour to ensure it is normal (as mentioned in Materials and Methods), BP fluctuations are not as significant as in patients who are not closely monitored. Smaller fluctuations of BP might have more impact on capillaries rather than larger blood vessels. Affecting large blood vessels may lead to early postoperative hematoma expanding or ischemia areas increasing, while affecting capillaries will generally decrease the patient's cognitive function, prolong the recovery period, and worsen the long-term prognosis [24–26, 28]. This maybe the main cause to explain why BP variability is only related to long-term outcomes, but not to short-term prognosis. Besides, in our study, “short-term outcome” was defined as whether patients suffered from recurrent strokes within 30 days after revascularization surgery. Mild damage caused by slight BP variability may not be detected by follow-up CT. We did not classify these invisible patients into the poor short-term prognosis group, leading to bias in the results.

Practically, the fluid management is part of the BP management. Patients with MMD can be hypotensive because preoperative fasting and intraoperative fluid loss often leave them in a state of dehydration [1, 8, 14, 23]. Furthermore, the metabolism of residual anesthetics and the use of mannitol to improve brain edema will exacerbate early postoperative patients' insufficient fluid volume. Fluid management is an easily overlooked issue, and the current articles on systematic assessment of fluid management are limited [23, 29]. Although we did not find that fluid management is related to patient's prognosis, it is reasonable to speculate a higher Hb level leading to a worse mRS grade is caused by blood concentration and insufficient fluid volume. In other words, insufficient fluid volume in the early postoperative period might be the contributor to the worse prognosis of patients with elevated Hb level. Hemodilution is a way of improving cerebral perfusion and avoiding cerebral ischemia. Actually in our cohort, to avoid hypotension and hypovolemia, each patient was given an intravenous administration of extracellular fluid prophylactically before and after operation.

Except that higher postoperative Hb levels after surgery were suspected to cause a worse long-term prognosis of patients, other RBC-related parameters have little effect on patients during the perioperative period. The principal reason why Hb increased within the 24 h after operation in our study might be blood concentration as we mentioned before. Higher Hb level, higher hematocrit level, and blood concentration can cause increased plasma viscosity and platelet reactivity and may lead to cerebral infarction or cerebral venous thrombosis, although increasing hemoglobin can enhance the carrying capacity of brain oxygen [11, 20]. Moreover, a higher hematocrit level can also lead to intrinsic microcirculatory venous congestion at the site of the STA-MCA anastomosis [20]. Unfortunately, we did not detect the relationship between Hct and patient prognosis. In addition, the suspicious differences caused by hemoglobin disappeared after adjusting for gender and age. More researches are needed.

Our study had some limitations. First, this is a single-center and nonrandomized-controlled trial. Second, it is a retrospective study. Therefore, selection bias may have existed. Larger prospective studies are essential to further investigate how to minimize the incidence of complications for MMD patients after revascularization.

## 5. Conclusions

The perioperative hemodynamic factors may have a certain impact on the patients' quality of life after revascularization surgery in MMD. Both high postoperative MAP levels and large MAP variability might affect the short-term or long-term prognosis of patients with MMD. What is more, we also found that a higher postoperative Hb level might be related with a worse outcome. These findings need to be confirmed by further research with larger sample populations. Accordingly, exploring an individualized hemodynamic strategy and possibly forming a comprehensive predicting system for perioperative management could be promising to achieve a better outcome for patients with MMD.

## Figures and Tables

**Table 1:** Demographics and baseline characteristics of patients with MMD.

Variable	Value
Age, y (mean ± SD)	43.8 ± 11.4
Sex, male (Num. (%))	171 (49%)
Diagnosis	
Moyamoya disease (Num. (%))	307 (88%)
Moyamoya syndrome (Num. (%))	40 (12%)
Clinical type	
Ischemic (Num. (%))	229 (72%)
Hemorrhagic (Num. (%))	87 (28%)
NIHSS at onset (mean ± SD)	2.7 ± 3.6
NIHSS at admission (mean ± SD)	1.5 ± 2.2
Suzuki stage	
I (Num. (%))	0 (0%)
II (Num. (%))	11 (3.2%)
III (Num. (%))	92 (26.5%)
IV (Num. (%))	143 (41.2%)
V (Num. (%))	101 (29.1%)
VI (Num. (%))	0 (0%)
Laboratory findings	
Cholesterol (mean ± SD)	3.8 ± 0.9
LDL (mean ± SD)	2.4 ± 0.9
TG (mean ± SD)	1.8 ± 1.1
Glu (mean ± SD)	5.9 ± 1.8

Glu: glucose; LDL: low-density lipoprotein; MMD: moyamoya disease; NIHSS: National Institute of Health Stroke Scale; TG: triglyceride.

**Table 2:** Characteristics of perioperative hemodynamic parameters grouped by short-term stroke events and long-term mRS.

Variable	Short-term stroke events				Long-term mRS		
	1 (*n* = 179)	2 (*n* = 123)	3 (*n* = 45)	*p* value	Favorable (*n* = 270)	Unfavorable (*n* = 31)	*p* value
Preoperative MAP (mmHg)	95.3 ± 10.6	97.1 ± 10.6	98.2 ± 12.1	0.18	96.1 ± 10.9	97.4 ± 10.2	0.52
Preoperative RBC parameters							
Hb (g/L)	134.8 ± 16.3	133.7 ± 18.0	135.0 ± 13.7	0.81	133.6 ± 16.7	139.7 ± 13.7	0.06
Hct (%)	40.6 ± 4.2	40.1 ± 4.8	40.5 ± 3.8	0.68	40.2 ± 4.4	41.2 ± 3.6	0.21
RBC (×10^12^)	4.5 ± 0.5	4.5 ± 0.5	4.5 ± 0.4	0.77	4.4 ± 0.5	4.6 ± 0.4	0.23
Postoperative MAP (mmHg)	95.7 ± 11.5	97.5 ± 12.3	99.5 ± 10.9	0.11	95.7 ± 11.4	103.6 ± 10.4	<0.001^∗∗^
Postoperative RBC parameters							
Hb (g/L)	118.7 ± 16.1	116.7 ± 19.7	120.2 ± 14.5	0.43	116.9 ± 17.1	123.7 ± 13.0	0.03^∗^
Hct (%)	35.9 ± 5.1	35.4 ± 10.4	36.4 ± 4.0	0.73	35.4 ± 7.9	37.1 ± 3.8	0.25
RBC (×10^12^)	4.3 ± 3.2	4.2 ± 3.5	4.0 ± 0.4	0.84	4.3 ± 3.5	4.1 ± 0.4	0.75
Variability of MAP (%)	1.0 ± 12.9	0.9 ± 13.4	2.2 ± 12.9	0.84	0.26 ± 13.2	7.2 ± 13.5	0.006^∗^
Variability of RBC parameters							
Hb (%)	11.9 ± 10.3	12.6 ± 9.2	10.5 ± 8.1	0.46	12.3 ± 8.7	11.1 ± 8.9	0.47
Hct (%)	−11.9 ± 12.7	−13.1 ± 25.6	−11.8 ± 15.7	0.84	−12.5 ± 19.1	−9.7 ± 9.3	0.41
RBC (%)	−8.6 ± 58.8	−7.6 ± 69.3	−12.0 ± 69.3	0.91	−7.6 ± 66.3	−10.3 ± 8.9	0.82
Fluid transfusion during the first 24 h in NICU (ml)	2674 ± 777.4	2688 ± 640.9	2905 ± 743.9	0.15	2702 ± 744.2	2887 ± 726.7	0.19
Total urine volume during the first 24 h in NICU (ml)	2099 ± 678.9	2073 ± 567.7	2305 ± 694.7	0.11	2093 ± 648.5	2368 ± 748.2	0.02^∗^
Fluid balance during the first 24 h in NICU (ml)	574 ± 564.4	614 ± 583.1	600 ± 512.7	0.84	608 ± 583.1	519 ± 494.4	0.41

^∗^ *p* < 0.05; ^∗∗^*p* < 0.001. Hb: hemoglobin; Hct: hematocrit; NICU: neurological intensive care unit; mRS: modified Rankin Scale; MAP: mean artery pressure; RBC: red blood cell.

**Table 3:** Logistic regressions of perioperative factors associated with short-term stroke events and long-term mRS.

	Short-term stroke events		Long-term mRS	
	Adjusted model^†^	*p* value	Adjusted model^†^	*p* value
Preoperative MAP	0.020 (0.000, 0.041)	0.06	1.007 (0.973, 1.042)	0.69
Preoperative RBC parameters				
Hb	-0.004 (-0.022, 0.014)	0.65	1.028 (0.995, 1.064)	0.10
Hct	-0.032 (-0.097, 0.032)	0.32	1.047 (0.932, 1.175)	0.44
RBC	-0.312 (-0.891, 0.266)	0.29	1.670 (0.602, 4.631)	0.32
Postoperative MAP	0.024 (0.004, 0.044)	0.02^∗^	1.058 (1.022, 1.096)	0.001^∗^
Postoperative RBC parameters				
Hb	-0.006 (-0.022, 0.010)	0.50	1.025 (0.995, 1.055)	0.10
Hct	-0.010 (-0.039, 0.019)	0.49	1.008 (0.972, 1.045)	0.68
RBC	-0.025 (-0.095, 0.045)	0.48	0.952 (0.789, 1.149)	0.61
Variance of MAP	0.504 (-1.159, 2.167)	0.55	30.982 (2.112, 454.414)	0.01^∗^
Variance of RBC parameters				
Hb	-0.552 (-3.139, 2.035)	0.68	3.996 (0.048, 329.624)	0.54
Hct	-0.319 (-1.524, 0.887)	0.60	1.090 (0.205, 5.792)	0.92
RBC	-0.074 (-0.400, 0.251)	0.66	0.787 (0.265, 2.336)	0.67
Fluid transfusion during the first 24 h in the NICU	0.000 (-2.577, 0.001)	0.07	1.000 (1.000, 1.001)	0.11
Fluid balance during the first 24 h in the NICU	0.000 (0.000, 0.001)	0.48	1.000 (0.999, 1.001)	0.62

Data are presented as *β* (95% CI) in multivariate regression analyses ^†^adjusted for age (in continuous) and sex. ^∗^*p* < 0.05; ^∗∗^*p* < 0.001. Hb: hemoglobin; Hct: hematocrit; NICU: neurological intensive care unit; mRS: modified Rankin Scale; MAP: mean artery pressure; RBC: red blood cell.

## Data Availability

The data used to support the findings of this study are available from the corresponding author upon request.
